# Metastatic Extramammary Paget's Disease of Scrotum Responds Completely to Single Agent Trastuzumab in a Hemodialysis Patient: Case Report, Molecular Profiling and Brief Review of the Literature

**DOI:** 10.1155/2015/895151

**Published:** 2015-01-27

**Authors:** Peter Barth, Essel Dulaimi Al-Saleem, Kristin W. Edwards, Sherri Z. Millis, Yu-Ning Wong, Daniel M. Geynisman

**Affiliations:** ^1^Department of Hematology/Oncology Oncology, Fox Chase Cancer Center, Temple University Health System, Philadelphia, PA 19111, USA; ^2^Department of Pathology, Fox Chase Cancer Center, Temple University Health System, Philadelphia, PA 19111, USA; ^3^Department of Radiology, Fox Chase Cancer Center, Temple University Health System, Philadelphia, PA 19111, USA; ^4^Caris Life Sciences, Phoenix, AZ 85040, USA

## Abstract

Extramammary Paget's disease (EMPD) is a rare cancer. Although EMPD is usually noninvasive and treated with local therapy, once metastatic the prognosis of EMPD is poor and treatment options are limited. We report a case of a complete response to single agent trastuzumab in a hemodialysis patient with metastatic Her2/neu overexpressed EMPD of the scrotum. Molecular profiling of his case as well as 12 other EMPD and 8 mammary Paget disease (MPD) cases was completed and revealed multiple biomarker aberrations. Overexpression of Her2 was frequently noted (30%–40%) in both EMPD and MPD patients and when present can be effectively treated with Her2 targeted agents. Trastuzumab therapy can be safely utilized in a hemodialysis patient. In addition, multiple protein overexpression and loss were seen in EMPD including PD-1, PD-L1, PTEN, and AR as well as PIK3CA mutation. These findings may lead to possible therapeutic interventions targeting these pathways in a disease with few effective treatment options.

## 1. Background

Extramammary Paget's disease (EMPD) is a rare intraepithelial adenocarcinoma most often involving apocrine gland-bearing locations such as the vulva and perianal area [1]. Paget's disease was originally described affecting the areolar tissue of the nipple in 1874, and by 1889 the first case of EMPD of the scrotum was reported [2, 3]. The disease often presents as pruritic plaques with epidermal infiltration of Paget cells; invasive EMPD has a worse prognosis than the* in situ* variety [4, 5]. Treatment usually consists of local therapy such as wide excision, laser ablation, radiation, imiquimod, or 5-fluorouracil creams, but not infrequently the disease recurs [6]. EMPD has sometimes been associated with other malignancies such as colon, anal, renal, bladder, and prostate cancer and in recent surveillance, epidemiology, and end results (SEER) Program analysis and invasive EMPD were shown to lead to a 50% increased risk of developing a secondary malignancy [7, 8]. EMPD usually presents either with local or with locally advanced disease, but in the aforementioned SEER analysis, 2.5% of the time it presented with metastatic disease [7].

EMPD of the scrotum or penis (penoscrotal) is a less common variety and has been estimated to have an incidence of 1/3.7 million males annually [9]. Four recent case series of penoscrotal EMPD have highlighted its presentation, management, and outcomes [9–12]. Metastatic extramammary Paget's disease (EMPD) comprises a very small subset of EMPDs with no standard of care established. Interestingly, approximately up to 60% of EMPD has been shown to be HER2/neu positive with several individual reports of favorable response to therapy with trastuzumab and paclitaxel [13–15]. A single report of trastuzumab monotherapy in a woman with labial EMPD has been reported [16].

Herein we present a case report of a patient on hemodialysis with metastatic EMPD of the scrotum treated with single agent trastuzumab with a complete response. Targeted next generation sequencing (tNGS) was performed on his tissue and we also report molecular profiling of twelve additional EMPD cases and compare them to mammary Paget disease (MPD). The use of trastuzumab in hemodialysis patients is also discussed.

## 2. Case Presentation

This is a 71-year-old male with a notable medical history of EMPD, hypertension, and end-stage renal disease (ESRD) on hemodialysis who presented to our office for evaluation of a left neck mass.

**Figure 1:**
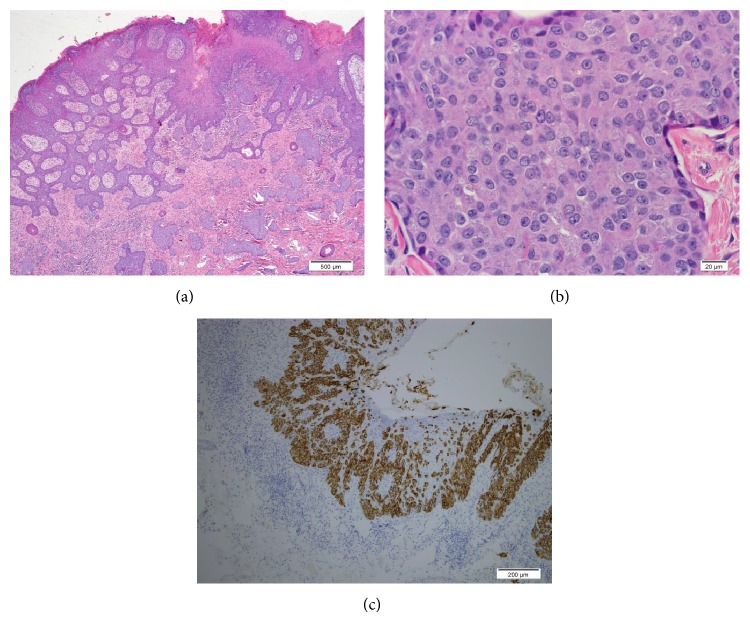
((a) and (b)) Skin and left scrotum involved by extramammary Paget's disease with invasive component: (c) cytokeratin 7 highlights adenocarcinoma cells.

His EMPD was initially diagnosed in 2011 when he noticed scrotal and perianal skin lesions. This was biopsied and pathology revealed EMPD with lymphovascular invasion and lateral margins positive for Paget's disease. Immunostaining was positive for CAM5.2, CK7, focally positive for BRST-2, negative for CK20, MART-1, and S100 (Figure 1). He was treated with surgical resection and reconstruction with a local flap. He then received adjuvant radiation to the affected area and did well for two years.

**Figure 2:**
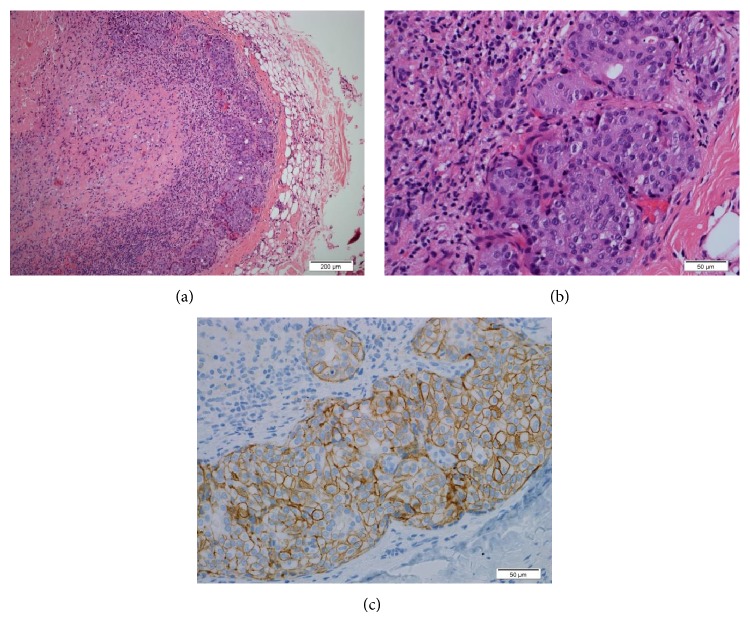
Lymph node H&E stain: (a) central necrosis tumor island in the subcapsular area, (b) adenocarcinoma cells with morphological features similar to Figure 1, and (c) complete membranous staining by HER2/neu immunohistochemical stain.

In 2013 he was referred to our institution following a work-up of an enlarged left neck mass which was biopsied and reported as metastatic adenocarcinoma of unknown primary with a broad differential diagnosis. The slides were examined in our institution which revealed that the lymph node tissue was entirely replaced by metastatic poorly differentiated adenocarcinoma associated with necrosis (Figure 2). A screening colonoscopy and upper endoscopy did not demonstrate any malignancy. The tumor cells by immunohistochemical stains were positive for CK7, CK17, CK19, CA19.9, CK5/6 (focal), PSMA (focal), and Ber-EP4 (focal) while they were negative for CDX2, CK20, p63, TTF-1, PSAP, and PSA. Tumor cells were also positive for BRST-2 (GCDFP). The morphological and immunohistochemical findings were consistent with metastatic EMPD.

Further immunohistochemistry revealed complete membranous positive HER2/neu staining (moderate to strong) in 90% of tumor cells. According to HER2 test guideline recommendations for breast cancer, this HER2 result would be considered to be positive (3+) (Figure 2). Next generation sequencing (NGS) on limited remaining lymph node tissue (FoundationOne) was performed, revealing two genomic alterations: DNMT3A R882H and SLIT2 rearrangement exon 37.

**Figure 3:**
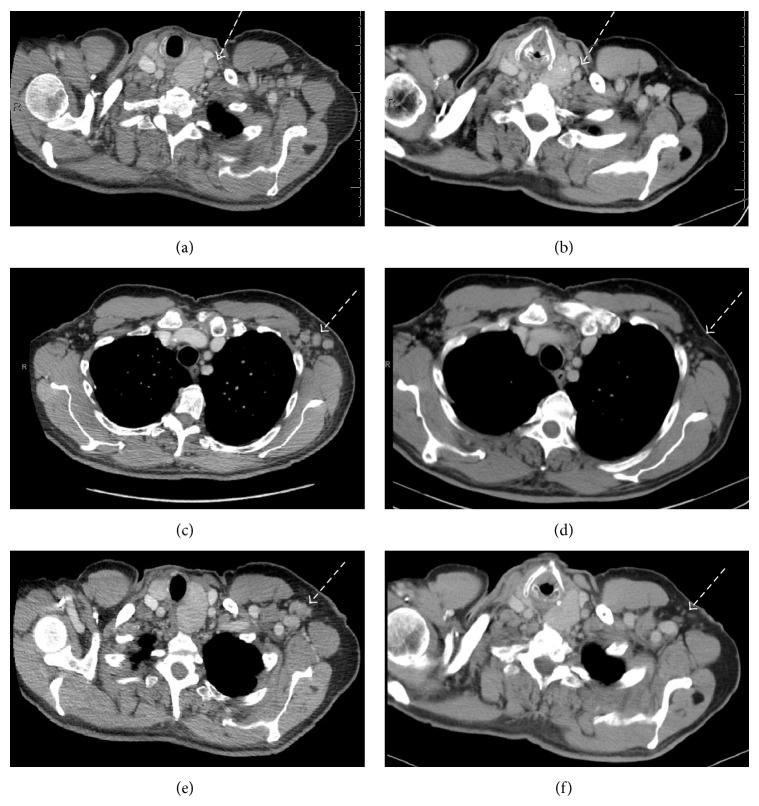
(a) Pretherapy enlarged left supraclavicular lymph node, (b) posttherapy normalized left supraclavicular lymph node, (c) pretherapy enlarged left axillary lymph nodes, (d) posttherapy normalized left axillary lymph nodes, (e) pretherapy enlarged left axillary lymph node, and (f) posttherapy normalized left axillary lymph node.

**Figure 4:**
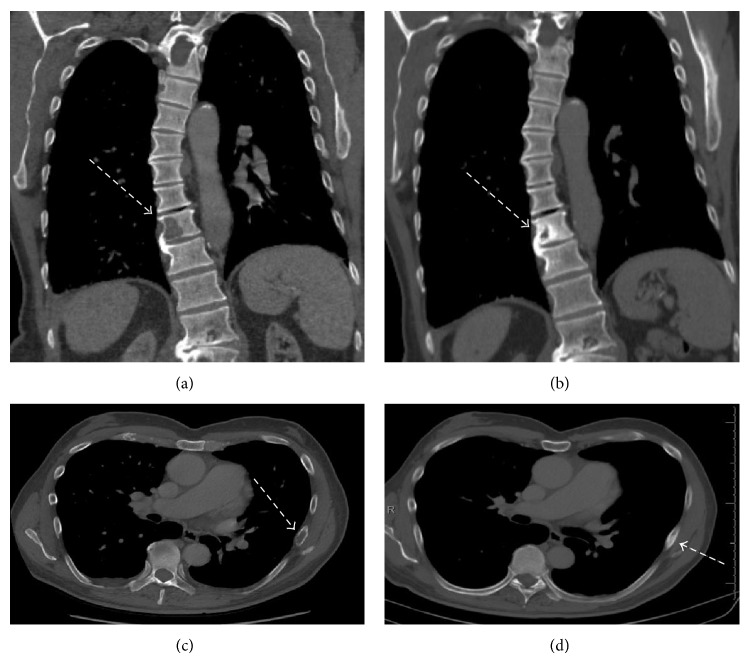
(a) Pretherapy T10 vertebral body lytic metastasis, (b) posttherapy T10 vertebral body becoming sclerotic, (c) pretherapy left 7th rib lytic metastasis, and (d) posttherapy left 7th rib met becoming sclerotic.

The patient underwent a diagnostic CT of the chest, abdomen, and pelvis which showed left cervical and axillary lymphadenopathy as well as a pathologic fracture through a lytic lesion of the left 7th rib and lytic lesion in the T10 vertebra (representative images of Figures 3 and 4).

Since he was on hemodialysis, it was decided to attempt to treat him with trastuzumab monotherapy rather than initiating cytotoxic chemotherapy, with a loading dose of 8 mg/kg followed by every third week treatment with 6 mg/kg, a standard dosing regimen for breast cancer. The patient tolerated therapy without any significant side effects, experiencing only mild fatigue for some days following each dose. Following the second treatment with trastuzumab, he had complete resolution of back pain that had been attributed to the T10 metastasis. Restaging CT after 4 doses or 3 months (Figures 3 and 4) revealed left axillary and left supraclavicular lymphadenopathy that had resolved, returning to normal size, consistent with treatment effect, and no new measurable disease in the chest, abdomen, or pelvis, and noted previously lytic bone metastases in the left seventh rib and spine now sclerotic, consistent with treatment effect. Almost 12 months after initiation of therapy, while undergoing periodic cardiac function analysis, the patient continues to receive trastuzumab therapy without significant side effects. Of note, he was diagnosed with squamous cell carcinoma under the right index nail bed in the midst of his treatment, approximately 3 months into treatment, and this was resected.

## 3. Conclusions

We present a case of a man with ESRD on hemodialysis with EMPD of the scrotum metastatic to the lymph nodes and bone, who underwent treatment with single agent trastuzumab and achieved a complete response by RECIST criteria. Our patient's case is of particular interest for two separate reasons: the use of trastuzumab to treat HER2/neu positive EMPD and the use of trastuzumab in a hemodialysis patient. Furthermore, we were able to characterize his disease further using NGS.

EMPD patients have in general a good prognosis due to the usual* in situ* only (epidermis only) spread of disease in which cases resection alone is often curative although a recurrence rate of 20–40% is observed in most series. Conversely, once being invasive into the reticular dermis or subcutaneous tissue or with lymph node or distant metastatic disease the prognosis is poor. Negative prognostic factors in EMPD include invasive disease, lymphovascular invasion, lymph node metastasis, and positive margins [17, 18]. In a series of 76 patients with EMPD, the overall 5-year survival was 85%, but 10/13 patients with metastatic disease died of EMPD [18]. In a series of 130 Chinese men with penoscrotal EMPD of whom 81 had long-term follow up, 5 (6%) died of metastatic disease [11]. A series of 20 penoscrotal EMPD patients from MD Anderson Cancer Center had a median OS of 14.5 months for those with invasive disease (*n* = 10), but only 3 died of actual EMPD, with several receiving neoadjuvant chemotherapy with docetaxel prior to surgery [9]. Interestingly, nine of the 20 patients had a second malignancy. A series of 25 Chinese and 19 South Korean penoscrotal EMPD patients showed only 1 and 2 deaths due to disease, respectively [10, 12].

Because of the rarity of metastatic EMPD, there are no clinical trials evaluating the best course of therapy. Cytotoxic chemotherapy described in case reports includes cisplatin, 5-fluorouracil, mitomycin C, paclitaxel, docetaxel, and epirubicin [19–21]. More recently, it has been observed that up to 50–60% of EMPD displays HER2 gene amplification and/or HER 2 protein overexpression [22, 23]. As a result two case reports of using trastuzumab with paclitaxel and a single report of single agent trastuzumab have been presented [13, 14, 16]. One patient had a response for 6 months and then progressed in the central nervous system, and the second received 6 cycles of therapy with disease stabilization but later required more local therapy and a third patient experienced a near complete response to single agent trastuzumab. No report of single agent trastuzumab in penoscrotal EMPD our knowledge has been presented and herein we report a second dramatic response to trastuzumab monotherapy in EMPD overall and the first in the setting of ESRD.

Interestingly, two gene alterations were noted in our patient: DNMT3A R882H and SLIT2. DNMT3A encodes a methyltransferase that is involved in the methylation of newly synthesized DNA. It is unclear as of yet if this is a tumor suppressor or an oncogene, but mutations are particularly seen in acute myeloid leukemia and myelodysplastic syndrome. DNA methyltransferase inhibitors include azacitidine and decitabine and this may be considered for an EMPD patient with this mutation. SLIT2 is a glycoprotein that binds receptors of the Robo family and provides guidance for cell migration and mutations have been seen in other aggressive tumors such as pancreatic cancer and small cell lung cancer. No approved therapies exist to target this mutation. ERBB2 overexpression or amplification was not detected in our patient on NGS as one may expect given the Her2/neu staining, but there was insufficient tissue to run a full analysis.

A recent case report described a man with metastatic EMPD which was ER+, but Her2/neu negative by IHC and FISH and who was treated initially with adriamycin, cyclophosphamide, and letrozole [24]. Due to NGS, a genomic ERBB2 S210F point mutation was found and the patient was successfully treated with lapatinib and capecitabine. Thus, to further investigate molecular profiling of EMPD, patient tissues submitted to Caris Life Sciences were examined using the methods previously published [25]. The sample size included 12 extramammary (anus *n* = 2, scrotum *n* = 1, and vulva *n* = 9) and 8 mammary Paget's disease cases. While not every test was performed on each tissue, comparison between EMPD and MPD identified differences in protein expression levels (immunohistochemistry), copy number (fluorescence* in situ* hybridization), and gene mutations (Sanger sequencing or next generation sequencing), as shown in Table 1. Hormone receptors ER and PR were overexpressed more frequently in mammary Paget's disease, although the single EMPD found in the scrotum had high expression of both ER and AR. Overexpression of HER2 was identified in 44% of EMPD of the vulva and 40% of MPD, which suggests that anti-HER2 agents are appropriate therapies in a subset of cases. PI3 kinase inhibitors may also be considered, as PIK3CA mutations were identified, in addition to loss of PTEN in 67% of MPD and 75% of EMPD. Anti PD-1 or PD-L1 therapy may also be useful given the overexpression noted in the scrotal case.

Finally, whether trastuzumab can be safely used in someone with ESRD on hemodialysis was not clear. Currently there are only two other reported cases of its use in ESRD [26]. One chronic hemodialysis patient with recurrent metastatic HER2 (3+) breast cancer was treated with trastuzumab without development of significant adverse effects. A second patient with ESRD also received trastuzumab in the adjuvant setting for invasive ductal carcinoma* in situ* and had clinical response without adverse effects. Serum concentrations of trastuzumab were not monitored in either of these cases. Our patient continued on hemodialysis without any additional side-effects.

Based on our case we recommend Her2 evaluation in every metastatic EMPD patient with the subsequent use of trastuzumab as first line therapy. The question remains whether the addition of paclitaxel or any other cytotoxic drugs adds to overall survival, progression-free survival, or response rate, but it is unlikely that this question will be answered given the rarity of the disease. Given the the importance of minimizing toxicity and optimizing quality-of-life in the treatment of a non-curable metastatic cancer such as EMPD, delaying cytotoxic therapy in favor of single agent trastuzumab may be prudent. In our patient on hemodialysis, trastuzumab was well tolerated and adds a third case in the published literature of its safe use in an ESRD population. We further characterized the molecular profile of EMPD and found evidence to support targeting several other well-known pathways in cancer.

## Figures and Tables

**Table 1:** Percent of cases by subtype with biomarker aberration and comparison of total EMPD versus MPD.

Primary tumor site	IHC																ISH			Sequencing	
	AR	BCRP	ER	Her2	MGMT	MRP1	PD-1	PD-L1	PDGFR	PR	PTEN^*^	RRM1^*^	TLE3	TOP2A	TOPO1	TS^*^	cMYC	EGFR	Her2	BRCA2	PIK3CA
Anus (*n* = 2)	0%	NT	0%	0%	0%	50%	NT	NT	NT	0%	50%	50%	NT	100%	0%	100%	NT	50%	NT	NT	NT
Scrotum (*n* = 1)	100%	NT	100%	0%	100%	NT	100%	100%	NT	0%	0%	0%	100%	100%	100%	0%	0%	NT	0%	100%	100%
Vulva (*n* = 9)	67%	67%	22%	44%	44%	75%	NT	NT	67%	11%	89%	67%	0%	44%	44%	89%	0%	0%	0%	NT	0%
All EMPD (*n* = 12)	**58%**	**67%**	**25%**	**33%**	**42%**	**70%**	**100%**	**100%**	**67%**	**8%**	**75%**	**58%**	**50%**	**58%**	**42%**	**83%**	**0%**	**11%**	**0%**	**100%**	**50%**
MPD (*n* = 8)	50%	33%	71%	36%	40%	75%	NT	NT	25%	43%	67%	100%	100%	20%	80%	100%	100%	0%	25%	NT	100%

Biomarker and technology used are shown for each subtype, as percent of total cases. A total of EMPDs are also compared to MPD. Percent in each box refers to overexpression of target proteins except for PTEN^*^, RRM1^*^, and TS^*^, where the percent refers to underexpression of the target. Genes in which no mutation was found: ABL1, AKT1, ALK, ATM, BRAF, BRCA1, BRCA2, CDH1, c-KIT, cMET, CSF1R, CTNNB1, EGFR, ERBB2, ERBB4, FBXW7, FGFR1, FGFR2, FLT3, GNA11, GNAQ, GNAS, HNF1A, HRAS, IDH1, JAK2, JAK3, KDR, KRAS, MLH1, MPL, NOTCH1, NPM1, NRAS, PDGFRA, PIK3CA, PTEN, PTPN11, RB1, RET, SMAD4, SMARCB1, SMO, STK11, TP53, and VHL.

IHC, immunohistochemistry; ISH, fluorescence in situ hybridization.
